# Vitellogenin Receptor as a Target for Tick Control: A Mini-Review

**DOI:** 10.3389/fphys.2019.00618

**Published:** 2019-05-21

**Authors:** Robert D. Mitchell, Daniel E. Sonenshine, Adalberto A. Pérez de León

**Affiliations:** ^1^USDA-ARS, Knipling-Bushland U.S. Livestock Insects Research Laboratory, Veterinary Pest Genomics Center, Kerrville, TX, United States; ^2^Laboratory for Malaria and Vector Research, National Institute of Allergy and Infectious Diseases, National Institutes of Health, Rockville, MD, United States; ^3^Department of Biological Sciences, Old Dominion University, Norfolk, VA, United States

**Keywords:** tick, vitellogenin receptor, vitellogenesis, RNAi, reproduction, tick-borne pathogens, vector control, vaccine

## Abstract

While much effort has been put into understanding vitellogenesis in insects and other organisms, much less is known of this process in ticks. There are several steps that facilitate yolk formation in developing oocytes of which the vitellogenin receptor (VgR) is a key component. The tick VgR binds vitellogenin (Vg) circulating in the hemolymph to initiate receptor-mediated endocytosis and its transformation into vitellin (Vn). The conversion of Vg into Vn, the final form of the yolk protein, occurs inside oocytes of the female tick ovary. Vn is critical to tick embryos since it serves as the nutritional source for their development, survival, and reproduction. Recent studies also suggest that pathogenic microbes, i.e., *Babesia* spp., that rely on ticks for propagation and dissemination likely “hitchhike” onto Vg molecules as they enter developing oocytes through the VgR. Suppressing VgR messenger RNA synthesis via RNA interference (RNAi) completely blocked *Babesia* spp. transmission into developing tick oocytes, thereby inhibiting vertical transmission of these pathogenic microbes from female to eggs. To date, VgRs from only four tick species, *Dermacentor variabilis*, *Rhipicephalus microplus*, *Amblyomma hebraeum*, and *Haemaphysalis longicornis*, have been fully sequenced and characterized. In contrast, many more VgRs have been described in various insect species. VgR is a critical component in egg formation and maturation that can serve as a precise target for tick control. However, additional research will help identify unique residues within the receptor that are specific to ticks or other arthropod disease vectors while avoiding cross-reactivity with non-target species. Detailed knowledge of the molecular structure and functional role of tick VgRs will enable development of novel vaccines to control ticks and tick-borne diseases.

## Introduction

Ticks are ectoparasites that blood feed on hosts found across diverse habitats ranging from the darkest caverns to the hottest deserts. They harbor a greater variety of pathogenic microbes, including bacteria, viruses, and protozoans, than any other arthropod group (Anderson and Magnarelli, 2008; Sonenshine and Roe, 2013a). Tick-borne pathogens are transmitted via the bite of an infected tick to a susceptible host. Successful transmission can result in debilitating or even lethal diseases like Lyme disease (causative agent *Borrelia burgdorferi*), Rocky Mountain spotted fever (causative agent *Rickettsia rickettsia*) and babesiosis (causative agent *Babesia* spp.) in humans, domesticated animals, and wildlife. Tick bites can also elicit a severe immune response as tick saliva carries a broad assortment of pharmacologically active molecules directed to inhibit host defenses (Alarcon-Chaidez et al., 2006; Nicholson et al., 2019; Nuttall, 2019). Ticks are masters of stealthy blood feeding and often remain undetected on an unsuspecting host for several hours or days as they feed. Hard ticks in the family Ixodidae typically feed for several days while soft ticks in the family Argasidae feed much more rapidly usually within minutes or only 1–2 h (Sonenshine and Roe, 2013b; Eisen, 2018). The injurious effects of tick bites and tick-transmitted diseases result in billions of dollars in damage annually to humans, livestock, and wildlife. Additional losses are incurred by the need to purchase and administer acaricides, medical and veterinary costs for treating affected humans and livestock, and other costs such as permanent damage to animal hides and reduction in meat quality and milk production from infested livestock (Giraldo-Ríos and Hurtado, 2018; Mac et al., 2019). Global climate change exacerbates these difficulties by increasing the habitable range of ticks, including Canada and Nordic countries. Current control measures continue to fail resulting in widespread resistance to multiple classes of acaricides (Bowman and Nuttall, 2008; Sonenshine, 2018; Boulanger et al., 2019). Discovering new or alternative targets to enhance or replace existing methods is required to maintain effective tick control efforts and vector-borne disease prevention.

Vitellogenesis is a critical mechanism in tick reproduction and a process that can be targeted for tick control. Vitellogenin (Vg) is synthesized in the fat body and midgut of a female tick after mating and transported through the hemolymph, captured by surface receptors called vitellogenin receptors (VgRs), and endocytosed into developing oocytes within the ovaries. Endocytosed Vg is transformed to vitellin (Vn), the functional form of the yolk protein found in oocytes, which provides nutrients essential for the developing embryos (Khalil et al., 2011; Xavier et al., 2018). VgRs, which are large transmembrane proteins of approximately 200 kilodaltons, serve as “gatekeepers” regulating the entry of Vg and pathogenic microbes discussed herein. Captured Vg is transported into developing oocytes via receptor-mediated endocytosis across clathrin-coated pits that are normally distributed evenly across a developing oocyte’s outer surface. Besides ticks, VgRs are found in vertebrates and other invertebrate organisms including crustaceans, and a wide variety of other arthropods. However, while they share key motifs critical to proper functionality, tick VgRs are different enough from VgRs of other organisms to be candidate vaccine targets (Roe et al., 2008; Kopáček et al., 2019).

Of the approximately 702 hard tick and 193 soft tick species (Guglielmone et al., 2010), only four tick VgRs have been successfully cloned and sequenced, namely, *Dermacentor variabilis*, *Rhipicephalus microplus*, *Amblyomma hebraeum*, and *Haemaphysalis longicornis* (Table 1). This is in stark contrast to the many more VgRs described in insect species. This marked disparity highlights the need for research on VgRs as targets to innovate tick control technologies. Advanced molecular techniques and next-generation sequencing can be applied to realize that potential. While the genomes of *Ixodes scapularis*, *Ixodes ricinus*, and *R*. *microplus* are now available, the genomes of other ticks of medical and veterinary importance must also be sequenced to further understand the role of VgRs in reproduction and tick-borne disease transmission to craft highly specific “designer molecules” for safer tick control (Cramaro et al., 2015; Gulia-Nuss et al., 2016; Barrero et al., 2017; Murgia et al., 2019).

**TABLE 1:** Tick vitellogenin receptor (VgR) sequence information available to date.

**Species**	**Common name**	**Protein name**	**Source**	**Acc.#**	**UniProt ID**	**Size^B^**
*Ixodes scapularis*	Black-legged tick; Deer tick	Vitellogenin receptor, putative	Genomic	EEC16350.1	B7QBX8	810
*Ixodes scapularis*	Black-legged tick; Deer tick	Vitellogenin receptor, putative	Genomic	EEC20133.1	B7QMR1	1200
*Rhipicephalus microplus*^A^	Southern cattle tick	Vitellogenin receptor	cDNA	AUQ44344.1	A0A2I7G3Y1	1799
*Rhipicephalus microplus*	Southern cattle tick	Vitellogenin receptor, partial	cDNA	AMZ04157.1	A0A1W5KSB7	788
*Amblyomma hebraeum^A^*	Tropical bont tick	Vitellogenin receptor	cDNA	AGQ57038.1	U5KCA6	1801
*Dermacentor variabilis^A^*	American dog tick	Vitellogenin receptor	cDNA	AAZ31260.3	Q45VP9	1798
*Haemaphysalis longicornis^A^*	Bush tick	Vitellogenin receptor, partial	cDNA	BAG14342.1	B1Q2W6	1781
*Ornithodoros erraticus*	European soft tick	Vitellogenin receptor	cDNA	–	A0A293LRH2	874
*Ornithodoros erraticus*	European soft tick	Vitellogenin receptor	cDNA	–	A0A293M1D3	1278
*Ornithodoros erraticus*	European soft tick	Vitellogenin receptor	cDNA	–	A0A293N0S6	459
*Ornithodoros erraticus*	European soft tick	Vitellogenin receptor	cDNA	–	A0A293LLZ8	640
*Ornithodoros erraticus*	European soft tick	Vitellogenin receptor	cDNA	–	A0A293LUB6	505
*Ornithodoros brasiliensis*	Mouro tick	Vitellogenin receptor	cDNA	–	A0A1D2AJA7	219
*Ornithodoros brasiliensis*	Mouro tick	Vitellogenin receptor	cDNA	–	A0A1D2AIF9	166
*Ixodes ricinus*	Castor bean tick	Putative vitellogenin receptor	cDNA	JAA65139.1	A0A0K8R3T2	140
*Ixodes ricinus*	Castor bean tick	Putative vitellogenin receptor	cDNA	JAC91887.1	A0A090X7C9	221
*Ixodes ricinus*	Castor bean tick	Putative vitellogenin receptor	cDNA	JAB68489.1	V5GWI7	133
*Ixodes ricinus*	Castor bean tick	Putative vitellogenin receptor	cDNA	JAA73497.1	A0A0K8RQX7	100
*Amblyomma cajennense*	Cayenne tick	Putative vitellogenin receptor	cDNA	JAC21573.1	A0A023FLL9	336
*Amblyomma cajennense*	Cayenne tick	Putative vitellogenin receptor	cDNA	JAC24396.1	A0A023FRH2	389
*Rhipicephalus appendiculatus*	Brown ear tick	Vitellogenin receptor	cDNA	–	A0A2D1UEP7	187
*Rhipicephalus appendiculatus*	Brown ear tick	Vitellogenin receptor	cDNA	–	A0A131Z1S4	286
*Ornithodoros turicata*	Relapsing fever tick	Putative vitellogenin receptor	cDNA	–	A0A2R5LQ71	265
*Rhipicephalus pulchellus*	Zebra tick	Vitellogenin receptor	cDNA	JAA55785.1	L7LVF0	286
*Ornithodoros moubata*	African relapsing fever tick	Vitellogenin receptor	cDNA	–	A0A1Z5L4E0	226
*Ornithodoros moubata*	African relapsing fever tick	Vitellogenin receptor	cDNA	–	A0A1Z5L2N2	79

*There are 26 sequences available between the National Center for Biotechnology Information (NCBI) and the Universal Protein Database (UniProt) that identify as whole or partial tick VgRs of which 4 have been functionally characterized (Accession numbers: AUQ44344.1; AGQ57038.1; AAZ31260.3; BAG14342.1).^A^Functionally characterized.^B^Length in amino acids.*

This mini-review addresses current knowledge of the structure and function of VgRs from the American dog tick, *D. variabilis*, the southern cattle fever or Asian blue tick, *R. microplus*, the tropical bont tick, *A. hebraeum*, and the Asian longhorned tick, *H. longicornis*. Suppression of tick VgRs is explored as a method to eliminate ticks as well as prevent transmission of tick-borne pathogenic microbes transovarially to the next generation. Further avenues of tick VgR research are also discussed.

## Tick Vitellogenin Receptor Structure and Function

**FIGURE 1:**
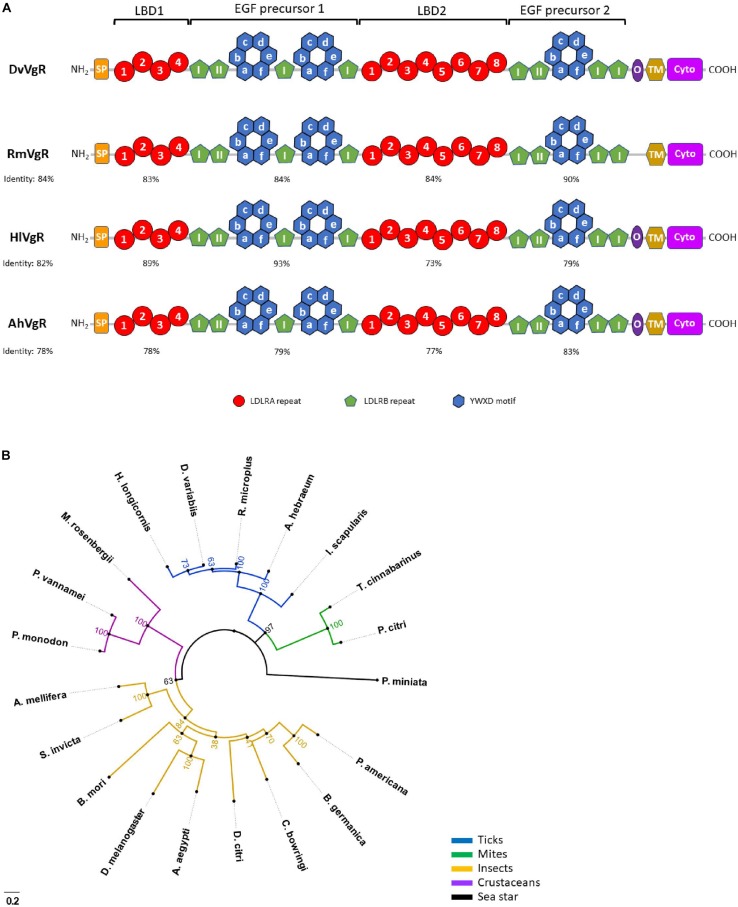
Tick vitellogenin receptor (VgR) structure and phylogeny in relation to VgRs from other organisms. **(A)** Tick VgRs from *D. variabilis* (DvVgR), *R. microplus* (RmVgR), *H. longicornis* (HlVgR), and *A. hebraeum* (AhVgR). Percentages under the species identifier represent the percent identity of that species with the full-length VgR amino acid sequence of *D. variabilis*. Percentages under the four highlighted domains represent the percent identity of that region in relation to the *D. variabilis* VgR amino acid sequence. LBD1, ligand-binding domain 1; EGF precursor 1, epidermal growth factor (EGF)-precursor homology domain 1; LBD2, ligand-binding domain 2; EGF precursor 2, epidermal growth factor (EGF)-precursor homology domain 2; “I” inside green pentagon, EGF-like repeat within LDLRB repeat of LBD (non-calcium binding); “II” inside green pentagon, EGF-like repeat within LDLRB repeat of LBD (calcium binding); SP, signal peptide; TM, transmembrane domain; O, O-linked sugar domain; Cyto, cytoplasmic domain. **(B)** Unrooted maximum likelihood tree showing the phylogenetic relationship between VgRs from 5 tick species (AAZ31260.3, *Dermacentor variabilis*; AGQ57038.1, *Amblyomma hebraeum*; AUQ44344.1, *Rhipicephalus microplus*; BAG14342.1, *Haemaphysalis longicornis*; EEC20133.1, *Ixodes scapularis*), 2 mite species (ANS13820.1, *Tetranychus cinnabarinus*; AHN48901.1, *Panonychus citri*), 9 insect species (AAK15810.1, *Aedes aegypti*; AAB60217.1, *Drosophila melanogaster*; BAC02725.2, *Periplaneta americana*; CAJ19121.1, *Blattella germanica*; AAP92450.1, *Solenopsis invicta*; XP_026295652.1, *Apis mellifera*; ADK94452.1, *Bombyx mori*; AZN28756.1, *Colaphellus bowringi*; XP_026689064.1, *Diaphorina citri*), 3 crustacean species (ADK55596.1, *Macrobrachium rosenbergii*; ROT71709.1, *Penaeus vannamei*; ABW79798.1, *Penaeus monodon*), and a sea star (AMR68937.1, *Patiria miniata*). Bootstrap values from 1000 simulations are displayed at the nodes. Number and letter combinations in parenthesis are Accession Numbers. Figure design modeled after figure from Smith and Kaufman (2013).

Tick VgRs, derived from a single gene, are members of the low-density lipoprotein receptor (LDLR) gene superfamily and share the common multi-domain architecture of other LDLRs (with a few exceptions discussed later) including: (1) ligand-binding domains (LBDs) consisting of clusters of cysteine-rich repeats, (2) cysteine-rich epidermal growth factor (EGF)-precursor homology domains, (3) an O-linked sugar domain, (4) a transmembrane domain, and (5) a cytoplasmic domain as shown in Figure 1A (Sappington and Raikhel, 1998; Tufail and Takeda, 2009). The two LBDs in ticks, where circulating Vg binds, consist of multiple modularly clustered cysteine-rich repeats, called LDLR class A (LDLRA) repeats, which are approximately 40 amino acids long and are disulfide-bonded in the pattern C_I_–C_III_, C_II_–C_V_, and C_IV_–C_VI_ (Goldstein et al., 1985). There is also a cluster of acidic residues in each repeat that generally follows the pattern (CDxxxDCxDGSDE), which is conserved between the fourth and sixth cysteines of all insect VgRs. This cluster of acidic residues within each LBD is critical for proper disulfide bond folding and ligand-binding to the domain (Blacklow and Kim, 1996; Fass et al., 1997). The LBD closest to the N-terminus, ligand-binding domain 1 (LBD1), in all tick VgRs described so far contains four LDLRA repeats while the LBD closest to the C-terminus, ligand-binding domain 2 (LBD2), contains eight, in contrast to the two LBDs found in insect VgRs where LBD1 contains five repeats while there are eight in LBD2. This difference contributes to insect LBD1 and LBD2 only having an approximately 35% identity with tick VgR LBDs (Boldbaatar et al., 2008; Smith and Kaufman, 2013).

In organisms less closely related evolutionarily to ticks, the number of repeats can vary substantially including vertebrate VgRs that have only a single 7- or 8-repeat LBD (Yamamoto et al., 1986; Okabayashi et al., 1996). As shown in Figure 1, LBD1 in *R. microplus*, *H. longicornis*, and *A. hebraeum* is 83, 89, and 78% identical (i.e., amino acids match exactly) to the *D. variabilis* LBD1, respectively. LBD2 in *R. microplus*, *H. longicornis*, and *A. hebraeum* is 84, 73, and 77% identical to the *D. variabilis* LBD2, respectively. This suggests that treatments targeting LBDs of tick VgRs may cross-react with multiple tick species while not targeting insects or other unrelated organisms. Additionally, there are essentially twice as many targets to interrogate in some of the more ancestral species since a second LBD seems to disappear in higher organisms. It is possible that genetic duplication occurred early in the molecular evolution of tick VgRs to generate new genetic material and was subsequently lost in higher organisms as VgR function became more specialized.

Tick EGF-precursor homology domains, which follow immediately after LBD1 and LBD2, contain four cysteine-rich LDLR class B (LDLRB) repeats in each domain. This contrasts insect VgRs that have four LDLRB repeats in the first domain but only three repeats in the second. However, unlike LDLRA repeats, LDLRB repeats follow the disulfide-binding pattern C_I_–C_III_, C_II_–C_IV_, and C_V_–C_VI_ (Sappington and Raikhel, 2005). There are also EGF-like repeats of approximately 40 amino acids that exist singly or in pairs within these domains. Some of these EGF-like repeats bind calcium to maintain stability of the domain and prevent proteolytic degradation (Figure 1A; Rao et al., 1995). Each tick EGF-precursor homology domain, as in other organisms, contains six YWXD motifs that form a β-propeller. The β-propeller is thought to function as an acid-dependent ligand release mechanism in endosomes but may also play an active role in ligand binding, although this has only been proposed in larger members of the lipoprotein receptor family (Andersen et al., 2013). However, not all algorithms pinpoint six YWXD motifs in this region in ticks and other organisms; research on this domain confirmed that the motif is not always absolutely conserved but there are always six repeats that must be present for functionality. Therefore, all β-propeller domains shown in Figure 1A are represented by six YWXD motifs even though they may have initially been reported as having less. The EGF-precursor homology domains may also serve as spacer regions to maintain adequate distance between LBDs and ensure that only ligands of certain sizes are retained by VgRs (Springer, 1998; Innerarity, 2002; Jeon and Blacklow, 2003; Andersen et al., 2013).

Smith and Kaufman (2013) reported only three EGF-like repeats in the EGF-precursor homology domain present following LBD1 in *H. longicornis*, but alternative algorithms, like the SMART algorithm, identify four EGF-like repeats in that domain (Smith and Kaufman, 2013; Letunic and Bork, 2018). The presence of three or four EGF-like repeats in that domain in *H. longicornis* is likely inconsequential as the basic elements are present for that domain to be fully functional in either scenario. The EGF-precursor homology domain closest to the N-terminus in *R. microplus*, *H. longicornis*, and *A. hebraeum* is 84, 93, and 79% identical to the same domain in *D. variabilis*, respectively (Figure 1A), while the EGF-precursor homology domain closest to the C-terminus in *R. microplus*, *H. longicornis*, and *A. hebraeum* is 84, 73, and 77% identical to the same domain in *D. variabilis*, respectively.

The O-linked sugar domain, a region rich in serine and threonine residues, is found in most, but not all, vertebrae and invertebrate VgRs. Research findings suggest that it may provide rigidity to the receptor as it extends into the extracellular space, afford protection from denaturation, or modulate proteolytic cleavage of the ectodomain (Gent and Braakman, 2004; Tufail and Takeda, 2009). The O-linked sugar domain is present in the VgR of *D. variabilis*, *H. longicornis*, and *A. hebraeum*, but is absent from the *R. microplus* VgR (Seixas et al., 2018). A hydrophobic transmembrane domain is present in all VgRs that tethers the receptor to the plasma membrane. Experiments disabling this region resulted in inactive, truncated receptors (Schneider and Nimpf, 2003). A cytoplasmic domain of all four tick species also contains typical leucine-leucine/leucine-isoleucine (LL/LI) and conserved FXNPXF sequences indicative of internalization signals in VgRs from other organism. These conserved residues play a critical role in receptor internalization as they are responsible for delivering ligand-bound receptors to internal endosomes where ligands are removed, and the receptors are recycled back to the cell surface (Trowbridge, 1991). Phylogenic analysis shows that ticks form a distinct clade when their VgRs are compared to those of mites, insects, crustaceans, and a sea star (Figure 1B). However, the functional significance of structural variations between tick VgRs and those of other invertebrates and vertebrates remains to be determined.

## Silencing Vitellogenin Receptor Halts Egg Formation and Deposition

RNA interference (RNAi) was utilized to ascertain the functionality of tick VgRs from *D. variabilis*, *H. longicornis*, *A. hebraeum*, and *R. microplus*. Mitchell et al. (2007) reported for the first time successful knockdown of a VgR in any acarine species. By disabling VgR messenger RNAs (mRNAs) the receptor was rendered ineffective and substantial amounts of Vg accumulated in the hemolymph of treated ticks rather than in the oocytes. Northern blot analysis revealed that VgR mRNAs from *D. variabilis* were abundant in the ovaries of vitellogenic females, but other female tissues and male whole-body extracts showed no VgR mRNAs. This observation revealed VgR expression to be tissue- and sex-specific. In these experiments, newly emerged unfed females were injected with 0.5 μg of double-stranded RNA (dsRNA) and placed on a rabbit host. These females were allowed to mate with introduced males and feed to repletion (∼8 days), then collected, and held 0–4 days post drop-off (when they were presumably fully vitellogenic, i.e., flooding nutrients into developing oocytes) before being dissected for assessment. In the PBS-injected control group the oocytes were almost completely brown from Vg uptake 2 days post drop-off (oocyte growth stage 4 as described in Balashov, 1972). In stark contrast, most of the RNAi-treated oocytes had not progressed past stage 2 in their development, which is typical of previtellogenic oocytes, the ovary was largely white in color, and mated females did not lay eggs.

Subsequently, *H. longicornis* (Boldbaatar et al., 2008), *A. hebraeum* (Smith and Kaufman, 2013), and *R. microplus* (Seixas et al., 2018) VgRs were sequenced, characterized, and shown to share structural features with the *D. variabilis* receptor. Table 1 shows all tick VgR sequences that are currently available in the NCBI and UniProt databases (UniProt Consortium, 2007; Lipman et al., 2015). Molecular studies were conducted to determine functionality and similar results were obtained to the *D. variabilis* work. *H. longicornis* females injected with 1.0 μg VgR-dsRNA did not lay eggs and their ovaries were predominantly white upon inspection 7 days post drop-off. In *A. hebraeum*, 1.0 μg VgR-dsRNA was injected into females and transcript suppression was observed, but blockage of Vg entering oocytes at the level of the previous studies was not achieved. It was suggested that this tick species may require an additional unknown vitellogenin uptake factor (VUF) for yolk uptake (Smith and Kaufman, 2013), but such a factor remains to be identified. The most recent tick VgR described was that of *R. microplus* where 8.0 μg VgR-dsRNA was injected into partially engorged females that were then artificially bloodfed for 28 h before dissection. RNAi treatment halted Vg uptake as in previous experiments and hatching rates were significantly reduced compared to controls. An interesting additional observation was made where part-fed ticks that reached a certain weight (>35 mg) were not significantly affected by VgR-dsRNA introduction, which suggested a developmental threshold for efficacy. Further studies need to determine the amount of dsRNA necessary to fully inhibit vitellogenesis and pathogen migration across the VgR in all tick species, the optimal time and methods of delivery, and whether or not a VUF is involved.

## Vitellogenin Receptor Physiology and Tick-Borne Pathogens

Transovarial transmission is an important process for the maintenance of tick-borne pathogens (Randolph, 1994). Silencing VgR in *H. longicornis* not only halted oocyte development, but it also blocked *Babesia gibsoni* transmission from the midgut into oocytes (Boldbaatar et al., 2008). *B. gibsoni* is a protozoan that causes canine babesiosis, a disease whose clinical manifestations in dogs can range from mild fever and lethargy to multi-organ failure and death (Solano-Gallego et al., 2016). This observation had not been previously described in ticks even though researchers knew the tick ovary played an important role in pathogen transovarial transmission. Hussein et al. (2019) demonstrated a similar finding in *R. microplus* where silencing the VgR blocked *B*. *bovis* transmission and inhibited ovary maturation. This study also reported that while >90% of the females in all test groups laid eggs, the egg masses from the VgR-dsRNA-treated group were misshapen, and weighed less than half (43 ± 3.36 mg) of the buffer-injected (121 ± 4.94 mg) and untreated groups (109 ± 4.32 mg).

## Microbial Hijacking of Vitellogenesis Through Vitellogenin Receptor

As noted in the preceding section, inhibiting the VgR of *R. microplus*, an arthropod of great economic importance to the livestock industry globally, blocked transmission of *B. bovis* to maturing oocytes just as *B. gibsoni* transmission was blocked by silencing the *H. longicornis* VgR. *B. bovis*, the causative agent of bovine babesiosis, causes millions of dollars of damage annually by destroying cow hides, disrupting meat production, and disturbing milk production (Bock et al., 2004). Pathogen transmission from adult to young at the VgR interface should be examined further for potential intervention to control tick-borne diseases. Recently, it was demonstrated that vertical transmission of the rice stripe virus (RSV) in *Laodelphax striatellus*, the small brown planthopper, occurs when RSV hitchhikes by binding to Vg entering developing oocytes through VgR-mediated endocytosis (He et al., 2018). A similar mechanism may have evolved among tick-borne pathogens for transovarial transmission to the progeny of infected gravid females. Additionally, it is hypothesized that ticks have more than one site of Vg synthesis, as was observed in *L. striatellus* (Rosell-Davis and Coons, 1989). Compelling evidence exists supporting tick Vg synthesis in the fat body, midgut, and some ovary tissue of various species (Thompson et al., 2007; Boldbaatar et al., 2010; Khalil et al., 2011; Smith and Kaufman, 2014; Ramírez Rodríguez et al., 2016). Only Vg from planthopper hemocytes bound RSV, which suggests a conformational difference whereby specific viral surface peptides could allow this interaction to occur (Huo et al., 2019).

## Tick Control Strategies Exploiting Vitellogenin Receptor

In addition to injection, RNAi can be delivered effectively orally or topically to ticks and other arthropods for control purposes (Killiny et al., 2014; Marr et al., 2014). Therefore, VgR-dsRNA could potentially be applied directly to ticks or potential hosts or, alternatively, delivered parenterally for systemic host protection. To improve transport and control release, organic nanocarriers like liposomes or inorganic nanoparticles could be employed to improve dsRNA survivability or target specific tissues or cellular compartments (Lombardo et al., 2019). The rapid development of genome editing technologies, like those based on CRISPR/Cas9 (Sun et al., 2017), present the opportunity for biotechnology approaches targeting tick VgRs.

Vaccine development utilizing recombinant tick VgRs should be thoroughly appraised to inhibit oocyte maturation, egg deposition, and pathogen transmission (Xavier et al., 2018). VgRs are desirable targets because tick species have a single gene copy and as far as we know they are expressed only in the ovary. Although tick VgRs appear inaccessible to specific host antibodies, it is known that ticks have a “leaky gut” whereby antibodies could reach, and bind concealed VgRs (Kemp et al., 1989; da Silva Vaz et al., 1996; Jeffers and Roe, 2008; Hope et al., 2010). “Concealed” antigens may not be part of the typical tick-host interaction but can still be exploited to elicit an anti-tick immunological response. However, bioengineering of the recombinant protein may be required to enhance immunogenicity and overcome the need for frequent booster vaccinations to continually stimulate the specific anti-tick immune response (Opdebeeck, 1994; Willadsen, 2008). Reverse vaccinology could be applied to develop VgR-based anti-tick vaccines whereby epitopes would be identified to produce subunit vaccines or by using such sequences to produce chimeras containing other relevant peptide sequences (Guerrero et al., 2012; Miller et al., 2012; Valle and Guerrero, 2018).

## Future Directions

Tick VgRs are candidates for innovative tick control technologies as they play a critical role in tick reproduction and the transovarial transmission of tick-borne pathogens. Conserved structural characteristics suggests that designer molecules targeting VgRs could be tick-specific. However, tick VgRs also share structural characteristics with other arthropod pests. Biotechnological advances offer the opportunity to exploit this feature to innovate control technologies that are safer for non-target species and other, more simplistic vaccines.

## Contribution to the Field Statement

While much effort has been put into understanding vitellogenesis in insects and other organisms, much less is known of this process in ticks. There are several steps that facilitate yolk formation in developing oocytes of which the VgR is a key component. The tick VgR binds Vg circulating in the hemolymph to initiate receptor-mediated endocytosis and its transformation into vitellin (Vn). The conversion of Vg into Vn, the final form of the yolk protein, occurs inside oocytes of the female tick ovary. Vn is critical to tick embryos since it serves as the nutritional source for their development, survivability, and ultimately for the continuation of the species. Recent studies also suggest that pathogenic microbes, i.e., *Babesia* spp., that rely on ticks for propagation and dissemination likely “hitchhike” onto Vg molecules as they enter developing oocytes through VgRs. Suppressing VgR messenger RNA synthesis via RNA interference (RNAi) completely blocked *Babesia* spp. transmission into developing tick oocytes, thereby inhibiting vertical transmission of these pathogenic microbes from female to eggs. Detailed knowledge of the molecular structure and functional role of tick VgRs enables biotechnological applications to innovate control technologies for integrated management of ticks and tick-borne diseases.

## Data Availability

No datasets were generated or analyzed for this study.

## Author Contributions

RM and APL conceived the idea. RM wrote the manuscript. APL and DS reviewed and revised the manuscript.

## Conflict of Interest Statement

The authors declare that the research was conducted in the absence of any commercial or financial relationships that could be construed as a potential conflict of interest.
